# Theoretical Study of the Geometry of Dibenzoazepine Analogues

**DOI:** 10.3390/molecules27030790

**Published:** 2022-01-25

**Authors:** Małgorzata Szymańska, Irena Majerz

**Affiliations:** Faculty of Pharmacy, Wroclaw Medical University, Borowska 211a, 50-556 Wroclaw, Poland; irena.majerz@umw.edu.pl

**Keywords:** 5*H*-dibenzo[b,f]azepine, 10,11-dihydro-5*H*-dibenzo[b,f]azepine, 5*H*-dibenzo[a,d][7]annulene, 10,11-dihydro-5*H*-dibenzo[a,d][7]annulene, molecular structure, aromaticity

## Abstract

The geometry of dibenzoazepine analogues—typical multifunctional drugs—was investigated to find the geometrical parameters sensitive to the substitution of the central seven-membered ring. Exploration of the crystal structure database (CSD) shows that the geometrical parameter sensitive to the substitution of the carbon atom distance of the central ring not included in the aromatic rings to the plane through the carbon atoms common for the central ring and the aromatic side rings. Presence of the double bond in the central ring was reflected in its partial aromaticity expressed by the HOMED parameter. Some derivatives of 5*H*-dibenzo[b,f]azepine with flat conformation of the central ring are characterized by mobility of the electron density comparable to the mobility in the aromatic side rings. Influence of the surrounding on the investigated compounds was confirmed by comparison of the optimized molecules and the molecules in the crystal state where the packing forces can influence the molecular geometry.

## 1. Introduction

**Scheme 1:**
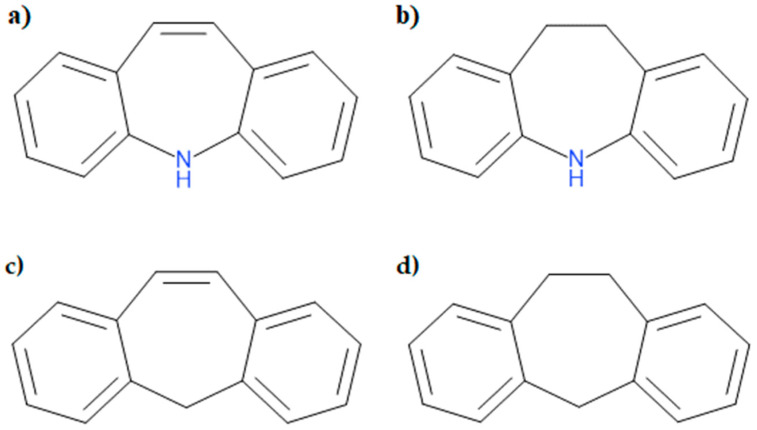
Structure of the investigated compounds: 5*H*-dibenzo[b,f]azepine (**a**), 10,11-dihydro-5*H*-dibenzo[b,f]azepine (**b**), 5*H*-dibenzo[a,d][7]annulene (**c**), 10,11-dihydro-5*H*-dibenzo[a,d][7]annulene (**d**).

The subject of this work are the compounds presented in Scheme 1. Their common feature is the central seven-membered ring with which two benzene rings are accumulated. The conformation of the middle ring is closely related to the presence of a double bond and the presence of a nitrogen or carbon atom in the 5-position. This group of compounds is important because many derivatives are used as medicaments. In a previous work we studied the geometrical and electronic structure of phenothiazines [1]—neuroleptic drugs acting as dopamine blocker. Phenothiazines are tricyclic compounds. Two side rings are aromatic, and the middle ring is aliphatic. It was important to investigate the effect of the substituents in the middle ring on the structure of the phenothiazines. In this work, we investigate similar tricyclic compounds, but the middle ring is seven-membered, which influences its antidepressant properties [2]. The first effective drug for such ailments was imipramine [3]. Thanks to the interest in this group of compounds, further 10,11-dihydro-5*H*-dibenzo[b,f]azepine derivatives were created [4,5,6] expecting them to be drugs as well. Navdeep Kaur synthesized a series of 10,11-dihydro-5*H*-dibenzo[b,f]azepine hydroxamates, which may have a positive effect on the treatment of cognitive vascular disorders [4].

Another important drug belonging to dibenzazepines is carbamazepine. Carbamazepine has anti-epileptic properties [7,8] and additionally relieves the pain [9]. It is used in the treatment of neuroleptic malignant syndrome [10]. Ruaa Wassim prepared a series of 1,2,3-triazole derivatives basing on *N*-acetyl-5*H*-dibenzo[b,f]azepine-5-carboxamide. One of these compounds showed an excellent activity against P. aeruginoa [11]. Kumar Honnaiah prepared a series of 5*H*-dibenzo[b,f]azepine derivatives to evaluate the structure-antioxidant activity relationship [12,13]. Promising results were obtained with 10-methoxy-5*H*-dibenz[b,f]azepine. The presence of the electron donating group OCH_3_ and the NH group in the middle ring may contribute to better antioxidant activity [12,13]. The derivative of 10,11-dihydro-5*H*-dibenzo[a,d][7]annulene is an antidepressant amineptine [14]. The interest in the derivatives of 10,11-dihydro-5*H*-dibenzo[a,d][7]annulene is quite large, as evidenced by numerous publications on the synthesis of new derivatives [15,16,17,18].

The last group of compounds which is worth attention are 5*H*-dibenzo[a,d][7]annulene derivatives with cytotoxic [19], antioxidant [20] and antimicrobial [21] properties. Kopanski confirmed effects of long-term treatment of rats with antidepressants on adrenergic-receptor sensitivity in cerebral cortex [22]. He observed that the sulfur or oxygen atom at the 10-position of dibenzocycloheptadienes (dibenzoazepine derivative) decreased the ability to induce down-regulation of the adrenergic receptor. He also noted that the effects of the drug were significantly influenced by changes in the chain substituted at the 5-position [22].

Because physicochemical and pharmaceutical properties as well as the mechanism of drug action in organisms are related to the molecular structure [12,13,23], we have undertaken a systematic theoretical study to analyze the structural parameters of 5*H*-dibenzo[b,f]azepine, 10,11-dihydro-5*H*-dibenzo[b,f]azepine, 5*H*-dibenzo[a,d][7]annulene and 10,11-dihydro-5*H*-dibenzo[a,d][7]annulene (Scheme 1). In the first step of the research, an analysis of the compounds available in the CSD crystallographic database [24] has been carried out. This analysis allowed for the determination of geometric parameters that change under substitution. The second step is comparison of the optimized structure with the experimental X-ray structure to check if the packing of the molecule in crystal can change the geometry of the molecule significantly. If so, it can be expected that also other factors resulting from the influence of the environment on the molecular geometry should be taken into account during the analysis of the environment of the drug in the living organism.

## 2. Computational Details

Geometries of the investigated compounds were retrieved from the 5.41 version of the CSD [24] with the updates in 2020. The search was performed without restrictions and gave 228 hits with 326 structures for 5*H*-dibenzo[b,f]azepine, 90 hits (126 structures) for 10,11-dihydro-5*H*-dibenzo[b,f]azepine, 428 hits (807 structures) for 5*H*-dibenzo[a,d][7]annulene and 277 hits (399 structures) for 10,11-dihydro-5*H*-dibenzo[a,d][7]annulene.

The investigated molecules were optimized using a Gaussian 16 package [25] at DFT-D3 B3LYP/6-311++G** level [26,27], with including Grimme dispersion [28]. DFT/B3LYP affords the best quality to predict the structure of organic compounds [29,30]. To check that the resultant geometry reached the energy minimum, vibrational frequencies were calculated. To visualize delocalization of electrons ACID program was used [31]. NBO analysis was performed using the ADF program [32,33,34].

## 3. Results and Discussion

### 3.1. Geometry of the Investigated Compounds

For 5*H*-dibenzo[b,f]azepine and 5*H*-dibenzo[a,d][7]annulene structures intramolecular proton transfer is possible [35,36]. For this purpose, the structures in Table 1 have been optimized. In order to decide which isomer 1 of 5*H*-dibenzo[b,f]azepine and 5*H*-dibenzo [a,d][7]annulene can exists in the investigated compounds, the energy of the isomers have been compared. The lowest energy structure indicates that, for the investigated compound, the isomer of the lowest energy is of a typical structure and the energy difference confirms that other isomers are not possible.

**Scheme 2:**
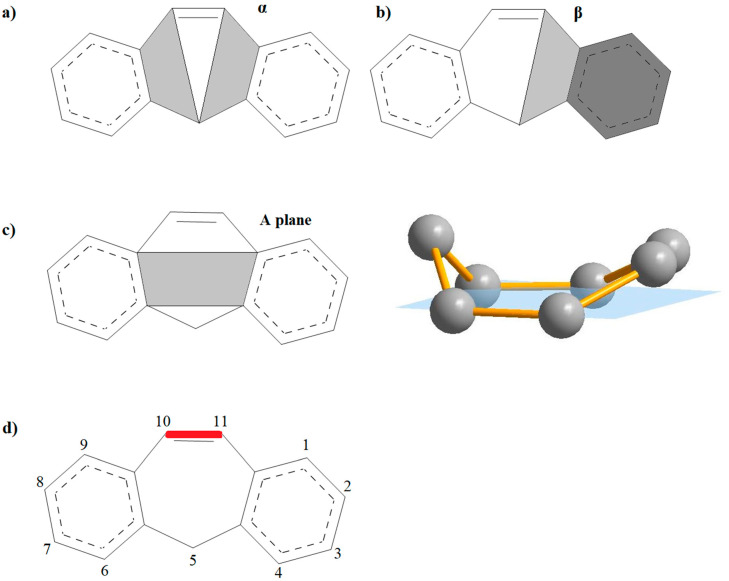
Geometric parameters for the analyzed compounds. α angle (**a**), β angle (**b**) (both angles are defined between the shaded planes). The A plane is defined by 4 carbon atoms in common with the side aromatic rings (**c**) (for clarity aromatic rings are not included), C10-C11 bond length (**d**).

The analysis of geometry of the investigated compounds should result from the indication of a geometric parameters that are sensitive to substitution and potential geometry changes in different environment of the molecule. Scheme 2 shows the geometric parameters which seem to be the most sensitive to substitution of the analyzed compounds, as follows: the α and β angle (both angles are between the shaded planes), the distances of the carbons and the heteroatom from the A plane defined by the carbons in the plane of the central ring shared with the aromatic rings and the C10-C11 bond length.

The results of the exploration of the CSD crystallographic base in relation to the above-mentioned geometric parameters are summarized in Table 2. The α angle for all the analyzed compounds does not reflect changes in geometry, because it changes slightly from 0 to 8 degrees for the analyzed compounds.

It can be expected that the C10C11 bond length should be typical for single or double CC bond. The data in Table 1 show that, depending on the substitution, the C10C11 bond can change in relatively wide range. In general, this bond is longer for azepine than for annulene derivatives.

**Figure 1:**
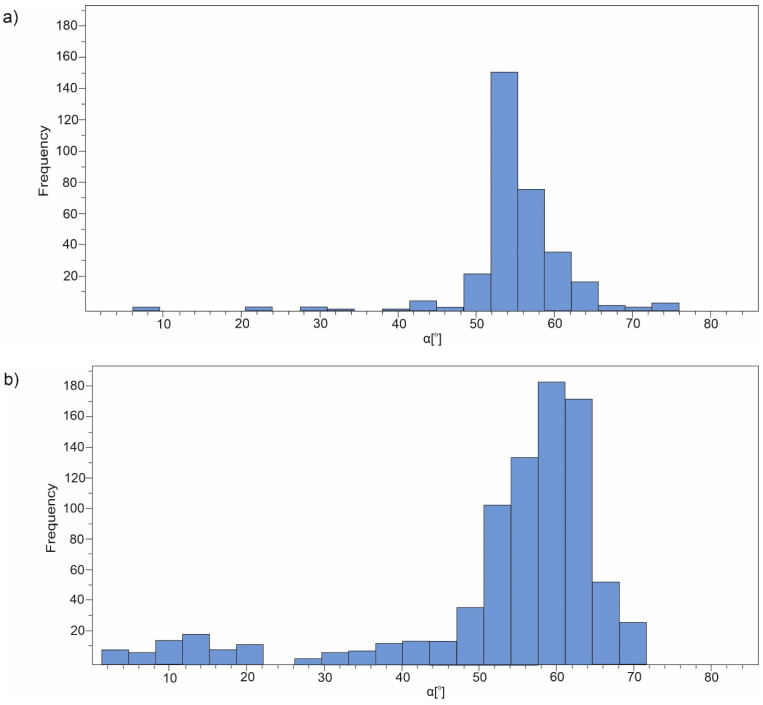

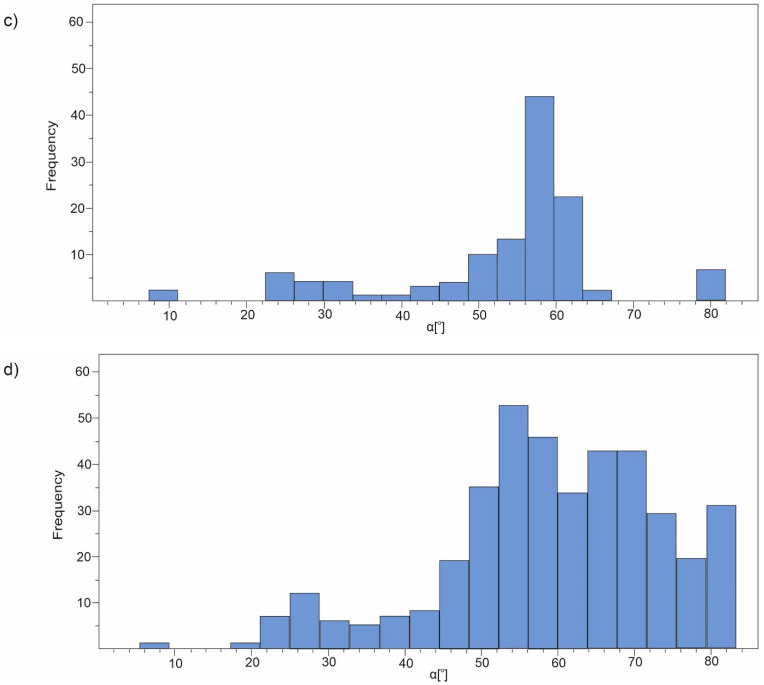
Histogram for the α angle of 5*H*-dibenzo[b,f]azepine (**a**), 5*H*-dibenzo[a,d][7]annulene (**b**), 10,11-dihydro-5*H*-dibenzo[b,f]azepine (**c**) and 10,11-dihydro-5*H*-dibenzo[a,d][7]annulene (**d**).

The histograms of the α angle performed for the compounds found in the CSD crystallographic database, as follows: 5*H*-dibenzo[b,f]azepine, 5*H*-dibenzo[a,d][7]annulene 10,11-dihydro-5*H*-dibenzo[b,f]azepine and 10,11-dihydro-5*H*-dibenzo[a,d][7]annulene are presented in Figure 1. The α angle covers a wide range of variation. For each group of compounds, the most frequent value can be detected except for 10,11-dihydro-5*H*-dibenzo[a,d][7]annulene. The α angle could be used as the parameter which describes nonplanarity of the central ring of the investigated compounds, but analysis of Table 2 suggests that the best geometrical parameters illustrating nonplanarity of the central ring are the distances of N, C5, C10 and C11 to the plane formed by the carbon atoms common with the aromatic rings (Scheme 2c).

According to the results in Table 2, the distances of the carbon and nitrogen atoms of the middle ring to the plane formed by the carbon atoms of the central ring shared with the aromatic rings vary widely. It is characteristic that very often the distance of these atoms from the plane is close to zero, which proves that the central ring becomes flat. Linear correlations between the distances of the C10 and C11 atoms from the A plane confirm the potential flattening of the middle ring. For 5*H*-dibenzo[b,f]azepine there is a straight line described by the following equation: y = 0.9217x + 0.0416, R^2^ = 0.8544. The mutual correlations of the distances from the plane of atoms C10 and N as well as C11 and N are described by a third-order polynomial, as follows: y = 5.5627x^3^ − 7.5677x^2^ + 3.822x − 0.2636, R^2^ = 0.6713 and y = 5.0552x^3^ − 6.9641x^2^ + 3.6504x − 0.2673, R^2^ = 0.6732, respectively. These correlations indicate that the shortening of the distances of the C10 and C11 atoms to the plane is coordinated, but not always associated with the placement of the nitrogen atom in the A plane.

Similar correlations exist for 5*H*-dibenzo[a,d][7]annulene. The correlation between the distance of C10 and C11 to the A plane is as follows: y = 0.9408x + 0.0291, R^2^ = 0.9067; the distance of C10 and C5 as well as C11 and C5 are as follows: y = 0.8678x − 0.0228, R^2^ = 0.7506 and y = 0.8693x − 0.0258, R^2^ = 0.7716, respectively.

**Figure 2:**
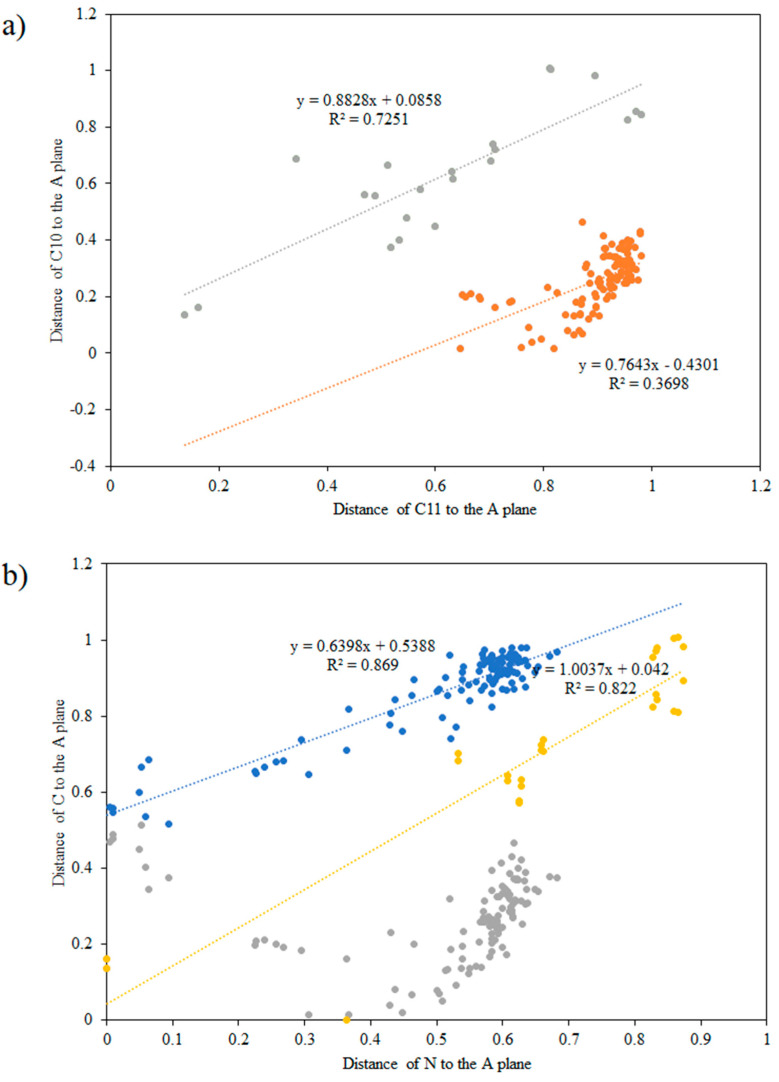
Correlation for 10,11-dihydro-5*H*-dibenzo[b,f][7]azepine. (**a**) Correlation of C10 and C11 distance to the A plane: gray—azepines with substituents in C10 and C11 positions, orange—azepines without substituents in C10 and C11 positions. (**b**) The distance of C10 and C11 to N5 of the A plane: blue and gray—azepines without substituents in C10 and C11 positions, yellow—azepines with substituents in C10 and C11 positions for 10,11-dihydro-5*H*-dibenzo[b,f][7]azepine.

While for 5*H*-dibenzo[a,d][7]azepine and 5*H*-dibenzo[a,d][7]annulene the distances of the carbon atoms to the A plane are similar, replacing of the double bond with a single one in 10,11-dihydro-5*H*-dibenzo[b,f]azepine leads to a difference in both distances. The replacement of the double bond with a single in 10,11-dihydro-5*H*-dibenzo[b,f]azepine causes that the correlation between the distance of C10 and C11 to the A plane can be detected for compounds with substituents at C10 and C11 atoms, while it is very weak for other compounds (Figure 2a). Differentiation of the C10 and C11 distance to the A plane results in different correlation lines for the distances for the N and C atoms. Additionally, the correlation of the longer C distance splits into correlation for substituted C10(C11) and unsubstituted. Correlation for shorter C distance to the A plane is not a straight line. The correlations in Figure 2 express irregular changes of C10, C11 and C5 distance to the A plane. For 10,11-dihydro-5*H*-dibenzo[a,d][7]annulene analogous correlations are not seen.

**Figure 3:**
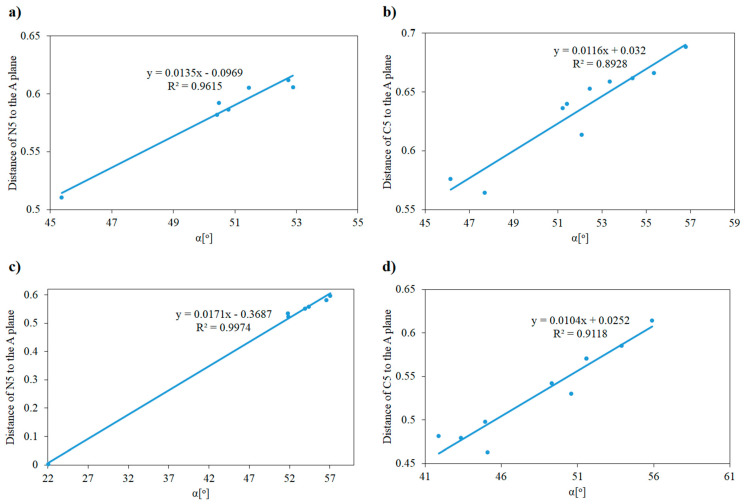
Correlation of the distance of the C5 or N5 atom from the A plane on the α angle on for: 5*H*-dibenzo[b,f]azepine (**a**), 5*H*-dibenzo[a,d][7]annulene (**b**), 10,11-dihydro-5*H*-dibenzo[b,f]azepine (**c**), 10,11-dihydro-5*H*-dibenzo[a,d][7]annulene (**d**).

To investigate the influence of substitution on the geometry of the 5*H*-dibenzo[b,f]azepine, 10,11-dihydro-5*H*-dibenzo[b,f]azepine, 5*H*-dibenzo[a,d][7]annulene and 10,11-dihydro-5*H*-dibenzo[a,d][7]annulene, the structures with CH_3_, CH_2_CH_3_, C(CH_3_)_3_, CHO, COOH, NO_2_, NH_2_, OH and Cl substituents in the central ring at the 5-position have been optimized. The values of α angle for the optimized structures change from 22 to 57^o^ (Table 3). An important parameter which, apart from the α angle, describes the non-planar structure of the molecule is the distance of the carbon and nitrogen atoms to the A plane. For this purpose, the α angle for the optimized compounds has been correlated with the distance of the C5 and N5 to the A plane (Figure 3). As the distance of the C5 and N5 atoms from the A plane increases, the α angle also increases and therefore flatness of the middle ring decreases. The shortest distance is observed for the nitrogen atom in 10,11-dihydro-5*H*-dibenzo[b,f]azepine without a substituent, which is also connected with the lowest α angle.

**Figure 4:**
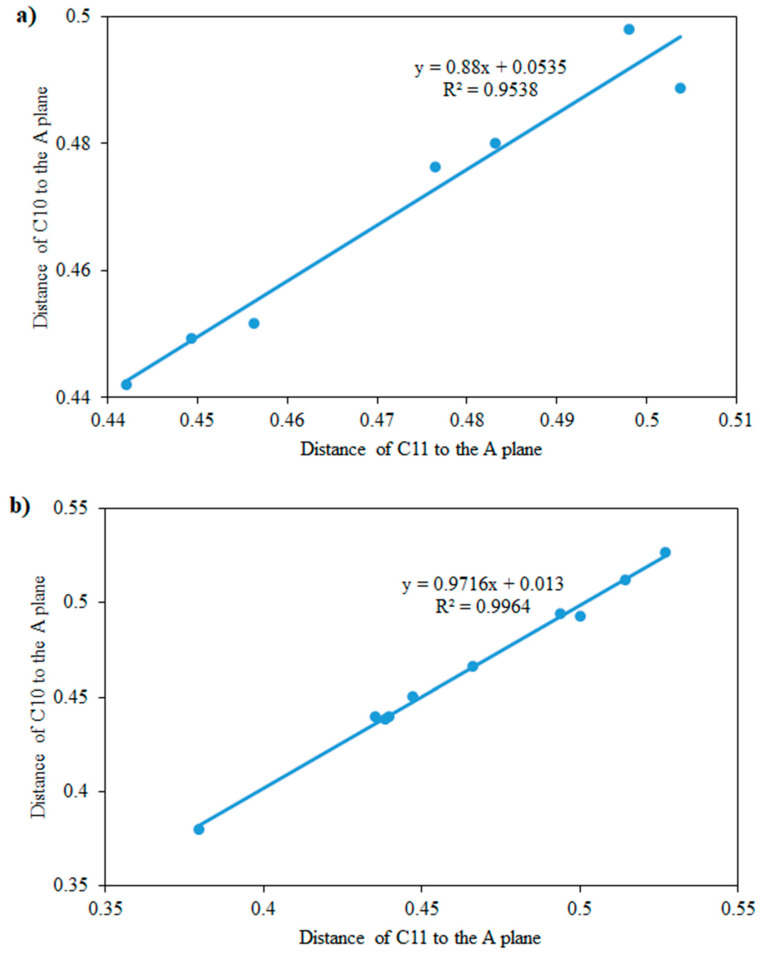
Correlation of C10 and C11 distance to the A plane for 5*H*-dibenzo[b,f]azepine (**a**), 5*H*-dibenzo[a,d][7]annulenne (**b**).

The distance of C10 and C11 to the A plane formed by the carbon atoms of the central ring common to aromatic rings has also been examined. The distances of C10 and C11 to the A plane are similar for both 5*H*-dibenzo[b,f]azepine and 5*H*-dibenzo[a,d][7]annulene derivatives. The analogous linear correlations exist for 5*H*-dibenzo[b,f]azepine y = 1.0838x − 0.0361, R^2^ = 0.9538, while for 5*H*-dibenzo[a,d][7]annulene y = 1.0255x − 0.0116, R^2^ = 0.9964 (Figure 4). The C10 and C11 distances to the A plane for 10,11-dihydro-5*H*-dibenzo[b,f]azepine and 10,11-dihydro-5*H*-dibenzo[a,d]annulene do not correlate with the α angle.

Despite sensitivity of the α angle to substitution of the investigated compounds, the C10C11 bond length changes slightly. The length of the C10C11 double bond in 5*H*-dibenzo[b,f]azepine and 5*H*-dibenzo[a,d][7]annulene changes from 1.345 Å to 1.352 Å. Larger differences from 1.529 Å to 1.544 Å are observed for the single C10C11 bond in 10,11-dihydro-5*H*-dibenzo[b,f]azepine and 10,11-dihydro-5*H*-dibenzo[a,d][7]annulene. In most cases the presence of nitrogen at the 5-position does not affect the length of the C10C11.

All analyzed changes in geometry indicate that the central ring in the investigated compounds is very flexible and may change the geometry from typical for completely aliphatic rings to almost flat. Changes in the geometry of the central ring result from its substitution. It can be also possible that the geometry changes can be caused by the environment of the molecule.

In order to study the influence of the environment, optimization of substituted structures of 5*H*-dibenzo[b,f]azepine, 10,11-dihydro-5*H*-dibenzo[b,f]azepine, 5*H*-dibenzo [a,d][7]annulene and 10,11-dihydro-5*H*-dibenzo[a,d][7]annulene has been carried out in solvents with different electric permittivity. The influence of the solvent on the α angle and the C10-C11 bond length has not been observed.

The structures of 5*H*-dibenz[b,f]azepine-5-carboxamide (carbamazepines) taken from the crystallographic database have been collected in Table 4. It is worth noting that carbamazepine has five polymorphs relating to the conformation of the middle ring, which is the reason for the differences in geometry [45]. The data in Table 4 have been compared with the optimized structure. The length of the C10C11 bond for the optimized molecule (1.350 Å) is very close to the median length of the same bond in the crystal structures. Similar results have been obtained for the distances of the C10 and C11 atoms from the A plane and for the optimized structure it is 0.491 and 0.506, respectively. For polymorphs it ranges between 0.347 and 0.650 for C10 and 0.395–0.570 for C11. Despite the fact that carbamazepine has a double C10C11 bond, the distances of C10 and C11 atoms from the A plane are different, which means that they do not lie in the same plane.

### 3.2. Aromaticity of the Central Ring of Investigated Compounds

Aromaticity is a phenomenon of the conjugated cyclic system of double bonds that shows delocalization of the π electrons. Such a system significantly modifies the chemical properties of the substances [53,54]. To determine the aromaticity of the rings of a chemical compound the Hückel’s rule is used. According to this rule, aromaticity is a property of conjugated, planar, cyclic compounds with 4n + 2 π-electrons where n is a natural number. Taking into account this rule, we have the following: for 5*H*-dibenzo[b,f]azepine the number of π electrons is 16 = 14 from 7(C=C) bonds + 2 from N lone pair; for 5*H*-dibenzo[a,d][7]annulene: 14 = 7 from (C=C) bonds; for 10,11-dihydro-5*H*-dibenzo[b,f]azepine: 14 = 12 from 6(C=C) bonds + 2 from N lone pair; for 10,11-dihydro-5*H*-dibenzo[a,d][7]annulene: 12 = from 6(C=C) bonds. According to the Hückel’s rule, aromatic compounds are: 5*H*-dibenzo[a,d][7]annulene and 10,11-dihydro-5*H*-dibenzo[b,f]azepine so the central ring for some of the investigated compounds must be almost flat if the term of planarity can be fulfilled. Because of the presence of the double bond in the central ring as well as the bonds common with the aromatic ring, conjugation of double bonds can be discussed. For 5*H*-dibenzo[b,f]azepine the lone pairs of the nitrogen atom may contribute to an increase in the aromaticity of the middle ring.

To describe and quantify aromaticity, many parameters resulting from geometry and physicochemical properties can be used [55,56,57,58,59,60]. The simplest and the most convenient to use, especially for large series of tested compounds, is the HOMA parameter basing on the bond length in the ring. For the benzene aromatic ring the HOMA index is equal to 1; for cyclohexane it is zero, for antiaromatic ring it is negative [61]. For compounds with heteroatoms in central ring, HOMED parameter is used, for which procedure, from a mathematical point of view, is the same as for HOMA, and CN parameter is included [35].

(1) $$\mathrm{HOMED}=1-\alpha /n\sum_{i=1}^{n}{\left(R_{\mathrm{opt}}-\;R_{\mathrm{ij}}\right)}^{2}$$

*R_opt_*—the optimized CC bond length of a perfectly aromatic system and equals 1.394 Å and the optimized CN bond length equals 1.334 Å

*R_ij_*—determined bond length

α—standardization constant: 5*H*-dibenzo[b,f]azepine for CN bond 84.52 and for CC bond 80.90, 5*H*-dibenzo[a,d][7]annulene for CC bond 80.90, 10,11-dihydro-5*H*-dibenzo[b,f]azepine for CN bond 73.20 and for CC bond 69.55, 10,11-dihydro-5*H*-dibenzo[a,d][7]annulene for CC bond 69.55

n—number of bonds

**Figure 5:**
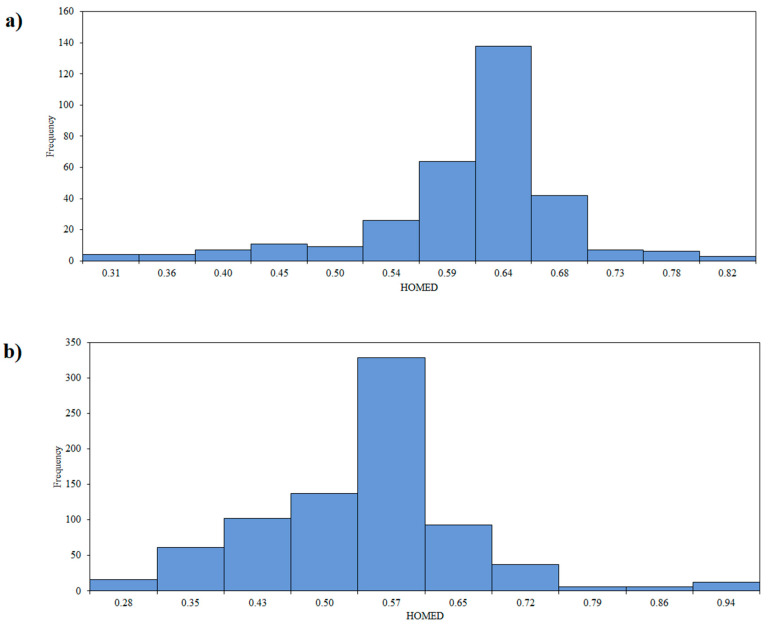

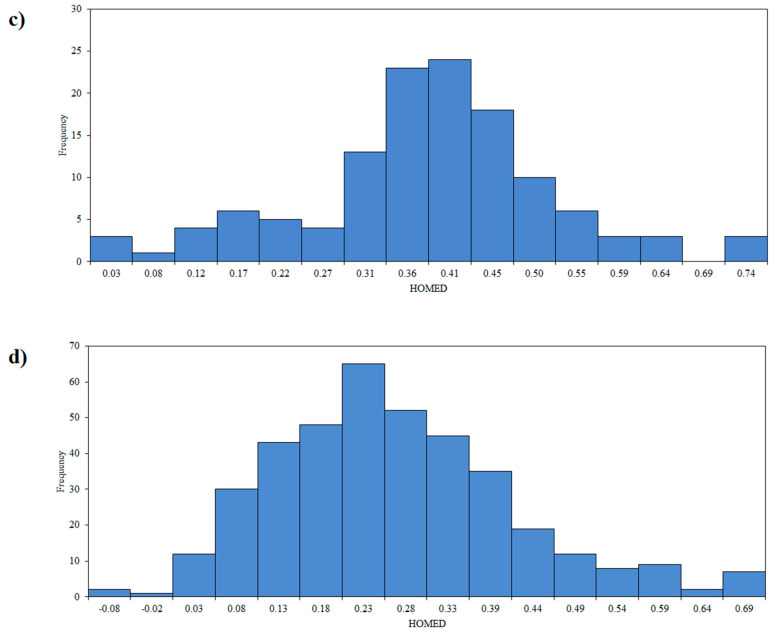
Histogram for the HOMED value for the middle ring of 5*H*-dibenzo[b,f]azepine (**a**), 5*H*-dibenzo[a,d][7]annulene (**b**) 10,11-dihydro-5*H*-dibenzo[b,f]azepine (**c**) and 10,11-dihydro-5*H*-dibenzo[a,d][7]annulene (**d**). Compound structures are taken from CSD crystal database.

Figure 5 shows histograms of the HOMED values for the middle ring of 5*H*-dibenzo[b,f]azepine, 10,11-dihydro-5*H*-dibenzo[b,f]azepine, 5*H*-dibenzo[a,d][7]annulene, 10,11-dihydro-5*H*-dibenzo[a,d][7]annulene taken from the database. According to the HOMED value for the central ring of 5*H*-dibenzo[b,f]azepine and 5*H*-dibenzo[a,d][7]annulene the ring is aromatic. The middle ring of 10,11-dihydro-5*H*-dibenzo[b,f]azepine and 10,11-dihydro-5*H*-dibenzo[a,d][7]annulene is less aromatic and the most frequent HOMED value is higher for 10,11-dihydro-5*H*-dibenzo[b,f]azepine than for 10,11-dihydro-5*H*-dibenzo[a,d][7]annulene.

**Figure 6:**
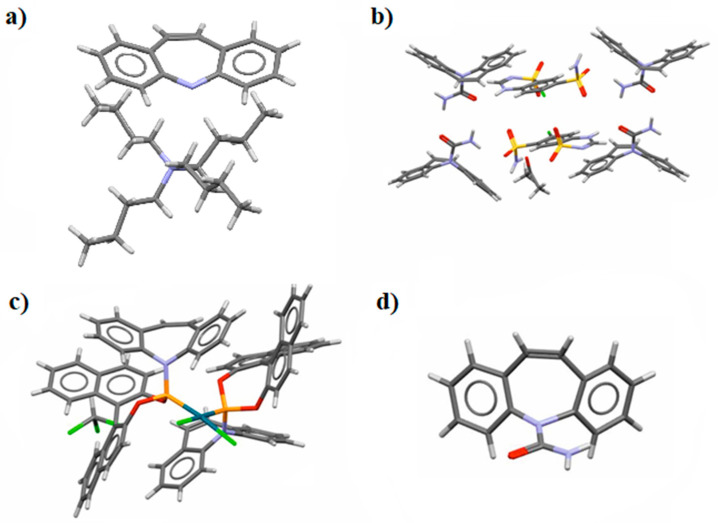
5*H*-dibenzo[b,f]azepine derivatives with the highest and the lowest HOMED values for the middle ring: YIJPEM [62] (0.8217) (**a**), VEJZUI [64] (0.7934) (**b**), YIFCUM [65] (−0.1731) (**c**), HEMRIB [63] (−0.2506) (**d**). In parentheses are given the HOMED values for the middle ring.

Comparison of the HOMED values for the derivatives of the investigated compounds listed in the CSD crystallographic database shows how much the aromaticity of the central ring depends on the substitution on the side rings and on the substituents in the central ring. While the HOMED value for the middle ring calculated for the optimized unsubstituted compound is 0.6876, substitution in both the middle ring and the side rings can lead to significant aromaticity changes. The highest HOMED value for the central ring of YIJPEM [62] is 0.8217, so this ring can be considered aromatic. The aromaticity of the central ring disappears in the case of HEMRIB [63] for which the HOMED value is −0.2506. In Figure 6 are presented the 5*H*-dibenzo[b,f]azepine derivatives with the highest and the lowest HOMED values for the middle ring.

The examples of 5*H*-dibenzo[b,f]azepine derivatives in Figure 6 with different HOMED values for the middle ring illustrate how the aromaticity of the central ring can be easily modified by the substituent and the environment of the molecule. This is especially true when comparing VEJZUI and HEMRIB. Despite the same substituent at the nitrogen atom, the middle ring can be aromatic or anti-aromatic depending on the surroundings of the molecule caused by crystal packing.

### 3.3. Delocalization of Electrons

**Figure 7:**
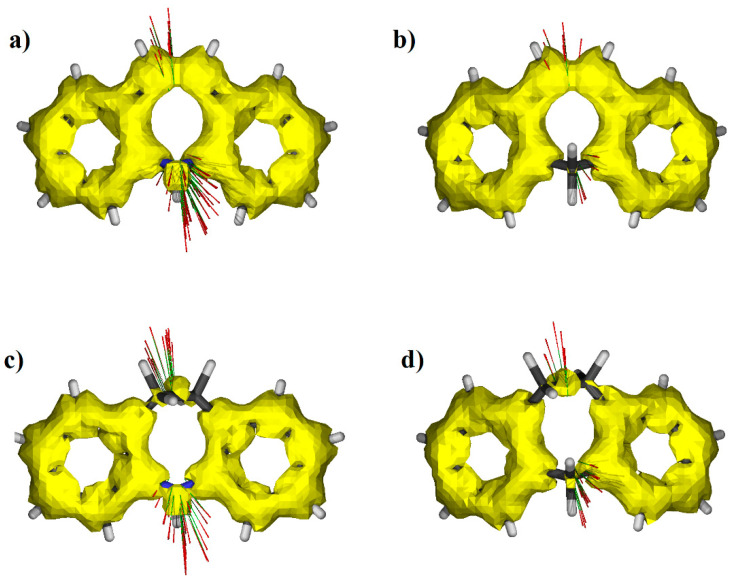
ACID surfaces for the optimized 5*H*-dibenzo[b,f]azepine (0.6876) (**a**), 5*H*-dibenzo[a,d][7]annulene (0.5448) (**b**), 10,11-dihydro-5*H*-dibenzo[b,f]azepine (0.4295) (**c**), 10,11-dihydro-5*H*-dibenzo[a,d][7]annulene (0.1863) (**d**). In parentheses are given the HOMED values for the middle ring.

The changes in aromaticity described by the HOMED parameter are closely related to the changes in the delocalization of the electron density which determines reactivity of the molecule and many other physical and chemical properties. A method to visualize the electron delocalization used in this work is ACID (anisotropy of the current-induced density) [31]. Delocalization of π electrons of the aromatic ring and the double bond is significant when comparing to delocalization of the single bond electrons, and this method allows indication of the bond character [66]. The ACID surfaces for the optimized structures of the investigated compounds are presented in Figure 7.

For the optimized compounds with double bond in the central ring, delocalization of the electrons is significant. The lone pair of the nitrogen in 5*H*-dibenzo[b,f]azepine participates in the mobility of the electrons of the central ring, so it has partially aromatic character expressed by the HOMED value of 0.6876. If nitrogen has been replaced by carbon, the lack of the lone electron pair prevents electron delocalization in 5*H*-dibenzo[a,d][7]annulene.

**Figure 8:**
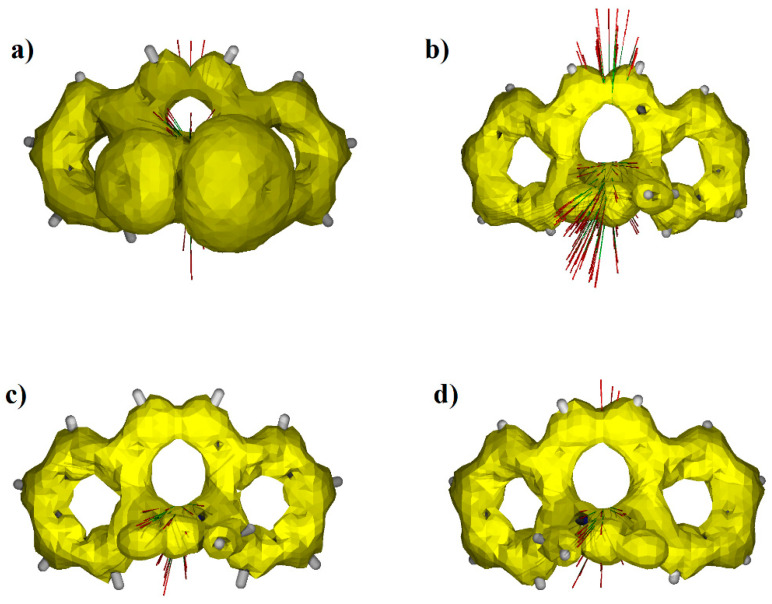
ACID surfaces for selected 5*H*-dibenzo[b,f]azepine derivatives with different aromaticity of the middle ring. The HOMED value for VUBCAW [67] is 0.7816 (**a**), for CBMZPN32 [52] −0.0896 (**b**), for TAZRAO01 [68] 0.6732 (**c**) and for HEMRIB [63] −0.2506 (**d**).

In Figure 8 are presented ACID surfaces for selected 5*H*-dibenzo[b,f]azepine derivatives. Because the HOMED values for the central ring can be higher than for the unsubstituted compound, delocalization of the electrons in the central ring can be similar to the aromatic side rings. For the antiaromatic central ring cumulated with two aromatic rings and with one double bond, antiaromaticity is expressed with breaking the continuity of electron delocalization at the aliphatic C-C bonds.

**Figure 9:**
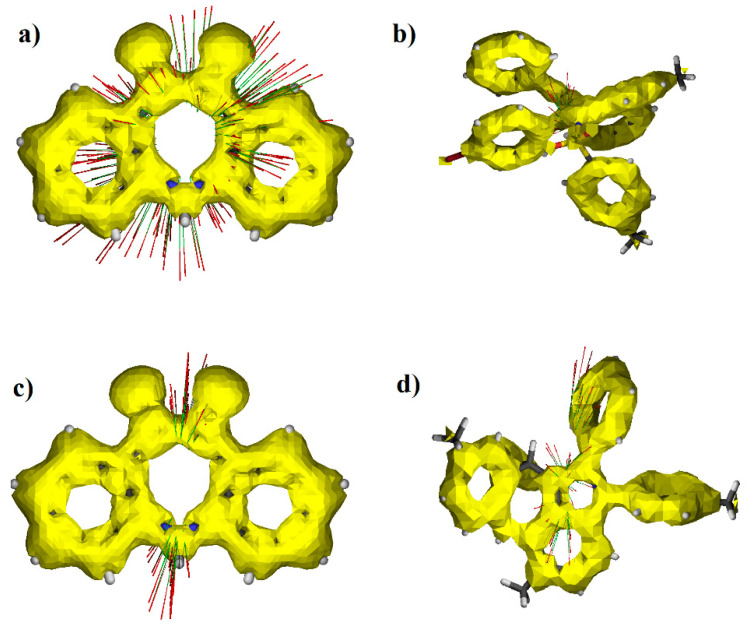
ACID surfaces for selected 10,11-dihydro-5*H*-dibenzo[b,f]azepine derivatives with different aromaticity of the central ring: GEXMAA01 [69] (0.7355) (**a**), RONRAQ [70] (0.7088) (**b**), GEXMAA [71] (0.6981) (**c**), TEVKUC [72] (0.6269) (**d**). In parentheses are given the HOMED values for the middle ring.

Replacing of the double bond in the middle ring with a single one caused the central ring to express less aromaticity. Substitution of the compound can cause the HOMED value for the middle ring to be higher than for a typical unsubstituted ring (Figure 9). Relatively high HOMED value and electron delocalization is connected with the presence of the lone pairs on the nitrogen atom and the aromatic bonds common for the central and the side ring.

**Figure 10:**
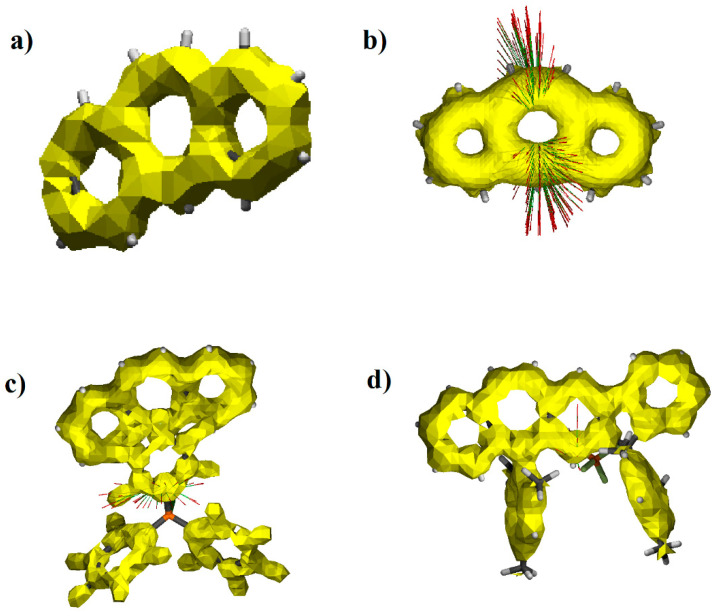
ACID surfaces for selected 5*H*-dibenzo[a,d][7]annulene derivatives with different aromaticity of the central ring: XOHXIC [73] (0.9363) (**a**), XOHXOI [73] (0.9201) (**b**), XOHXEY [73] (0.9169) (**c**), RULROI [74] (0.8818) (**d**). In parentheses are given the HOMED values for the middle ring.

Replacing of the nitrogen atom in the middle ring with a carbon atom reduces aromaticity and related electron delocalization comparing to the azepine. Nevertheless, appropriate substitution can change the nature of the central ring and the ring is not typically aliphatic (Figure 10). In order for the central ring to become typically aliphatic, it is necessary to replace the nitrogen with a carbon and replacing the double bond with a single bond.

### 3.4. NBO Analysis

Investigation of the chemical bond, especially the bonds in aromatic molecules, has a very long tradition. A particular chemical bond can be illustrated by molecular orbitals. To construct the molecular orbital representing the chemical bond, the natural atomic orbitals are transformed to natural atomic hybrid and finally to natural localized molecular orbitals (NLMO) which are close to molecular orbitals [75]. Natural localized molecular orbitals (NLMO) are traditionally used in chemistry to present the distribution of electron density in bonds linking atoms as well as in the lone pairs [76]. Detailed analysis of NLMO delivers information on participation of the atoms included in the bond, bond polarization, orbital occupancy and delocalization [77].

**Figure 11:**
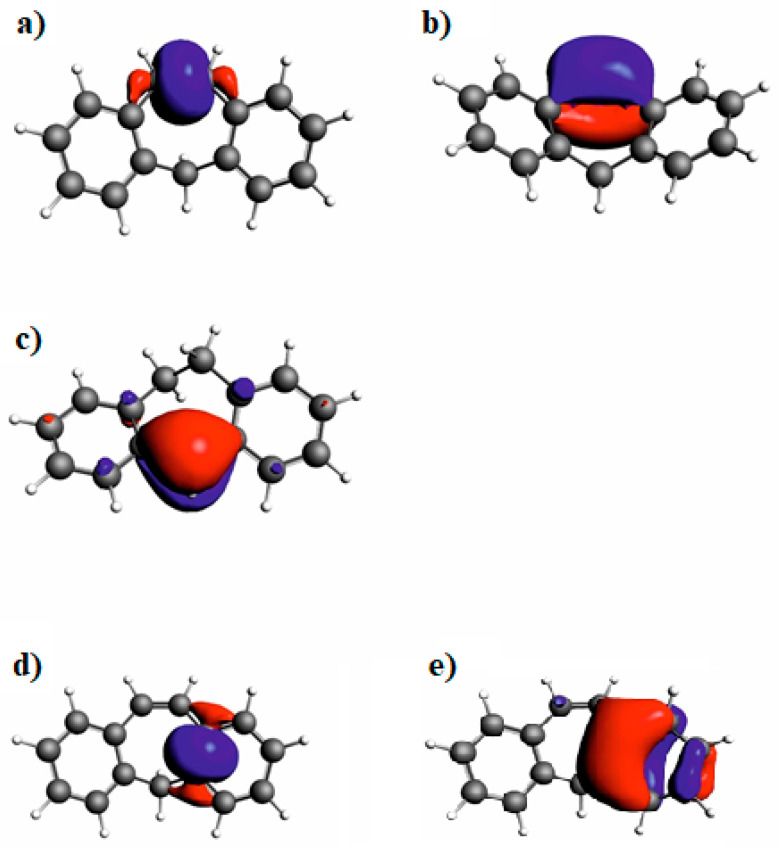
NLMO double bond molecular orbitals for optimized structure of 5*H*-dibenzo[a,d][7]annulene (**a**,**b**), lone pair on nitrogen atom orbitals for 10,11-dihydro-5*H*-dibenzo[b,f]azepine (**c**) and aromatic bond in 5*H*-dibenzo[a,d][7]annulene (**d**,**e**).

To explain the source of the partially aromatic character of the central rings of 5*H*-dibenzo[b,f]azepine and 5*H*-dibenzo[a,d][7]annulene the NLMO orbitals of this ring have been analyzed. In Figure 11 are shown the orbitals representing the double bond, the lone pair of the nitrogen atom and the aromatic bond common with the side ring. For a typical chemical bond, the localization is close to 100% and the occupancy is close to 2. One of the double bonds of 5*H*-dibenzo[b,f]azepine is localized and fully occupied (99.3329%, 1.9867). Occupancy of the second bond is 1.8823 when for the single bond it should be close to 2. Localization is 94.0865%, which is far off the normal localization of about 100%. The atoms next to the double bond also contribute to this bond, and their participation in the orbital is 1.3930%. It is characteristic that for 5*H*-dibenzo[a,d][7]annulene that localization, occupancy and participation of the neighboring atoms in the NLMO of the double bond is 93.5957%, 1.8733 and 1.563%, respectively.

The free electron pair on the nitrogen atom is also delocalized. For 5*H*-dibenzo[b,f][7]azepina, its location is 89.3911%, occupancy is 1.7909 and the participation of neighboring atoms is 1.192 and 0.822%, respectively. Free pair delocalization is more pronounced for 10,11-dihydro-5*H*-dibenzo[b,f]azepine.

Another source of delocalized electrons in the central ring are aromatic bonds in common with the side rings. During the NBO analysis, the aromatic side bond has been divided into one localized and fully occupied orbital and another with a location of approximately 79% and an occupancy of approximately 1.6000. The source of the partially aromatic character of the central rings of the investigated compounds is the delocalization of the free electron pair on the nitrogen atom, the delocalization of the double bond and the participation of aromatic electrons coming from the side rings.

## 4. Conclusions

The geometrical parameters that best describe the nonplanarity of the central ring of the investigated compounds are the distances of the C10, C11, and N (C) atoms in the 5-position from the A plane formed by carbon atoms common to the plane of the central ring and aromatic rings.

Although the central ring in 5*H*-dibenzo[b,f]azepine and 5*H*-dibenzo[a,d][7]annulene is not a typical aromatic ring, both the HOMED values and ACID diagrams indicate aromaticity of this ring for 5*H*-dibenzo[b,f]azepine and a significant participation of aromaticity in the case of 5*H*-dibenzo[a,d][7]annulene. The source of the partially aromatic character of the central rings of the investigated compounds is the delocalization of the free electron pair on the nitrogen atom, the delocalization of the double bond and the participation of aromatic electrons coming from the side rings.

## Figures and Tables

**Table 1:** Relative energies (ΔE in kcal × mol^−1^) for 5*H*-dibenzo[b,f]azepine and 5*H*-dibenzo[a,d][7]annulene isomers.

		Tautomer	ΔE [kcal۰mol^−1^]
5*H*-dibenzo[b,f]azepine	1	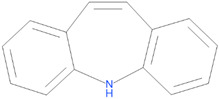	0
	2	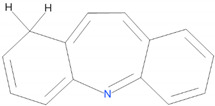	47.71
	3	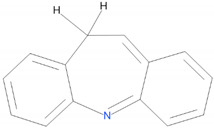	21.11
5*H*-dibenzo[a,d][7]annulene	1	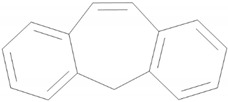	0
	2	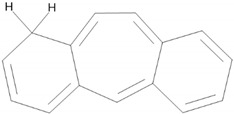	47.29
	3	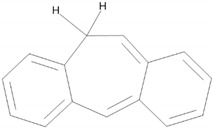	22.18

**Table 2:** Analyzed geometrical parameters for the investigated compounds.

Name	Minimum	Maximum	Mean	Variance	Std. Dev	Mean. Dev	Median
**α angle**							
5*H*-dibenzo[b,f]azepine	6.478	76.212	55.102	47.040	6.859	3.706	54.891
5*H*-dibenzo[a,d][7]annulene	1.196	72.078	54.257	192.096	13.860	9.081	58.611
10,11-dihydro-5*H*-dibenzo[b,f]azepine	7.035	82.424	53.577	180.502	13.435	9.313	57.400
10,11-dihydro-5*H*-dibenzo[a,d][7]annulene	5.028	83.931	59.767	206.198	14.36	11.258	60.169
**β1,β2 angle**							
5*H*-dibenzo[b,f]azepine	0.183	8.312	3.155	1.975	1.405	1.053	3.135
5*H*-dibenzo[b,f]azepine	0.273	9.489	3.117	2.428	1.558	1.178	3.107
5*H*-dibenzo[a,d][7]annulene	0.041	15.744	3.399	3.374	1.837	1.428	3.202
5*H*-dibenzo[a,d][7]annulene	0.044	11.373	3.449	3.161	1.778	1.41	3.251
10,11-dihydro-5*H*-dibenzo[b,f]azepine	0.416	19.911	3.094	6.373	2.525	1.627	2.42
10,11-dihydro-5*H*-dibenzo[b,f]azepine	0.441	15.258	2.967	5.078	2.253	1.508	2.266
10,11-dihydro-5*H*-dibenzo[a,d][7]annulene	0.136	12.738	3.433	4.439	2.107	1.608	3.062
10,11-dihydro-5*H*-dibenzo[a,d][7]annulene	0.072	12.512	3.21	4.658	2.158	1.636	2.736
**Distance to the C12,C13,C14,C15 plane**							
5*H*-dibenzo[b,f]azepine *C10*	0.004	0.884	0.537	0.008	0.087	0.056	0.527
5*H*-dibenzo[b,f]azepine *C11*	0.028	0.864	0.536	0.007	0.087	0.054	0.534
5*H*-dibenzo[b,f]azepine *N*	0.105	0.833	0.626	0.006	0.077	0.037	0.629
5*H*-dibenzo[a,d][7]annulene *C10*	0.002	1.003	0.525	0.025	0.158	0.114	0.556
5*H*-dibenzo[a,d][7]annulene *C11*	0.003	0.891	0.523	0.024	0.156	0.112	0.550
5*H*-dibenzo[a,d][7]annulene *C5*	0.005	0.818	0.632	0.025	0.158	0.101	0.677
10,11-dihydro-5*H*-dibenzo[b,f]azepine *C10*	0.000	0.873	0.532	0.034	0.184	0.125	0.587
10,11-dihydro-5*H*-dibenzo[b,f]azepine *C11*	0.015	0.980	0.554	0.103	0.32	0.295	0.514
10,11-dihydro-5*H*-dibenzo[b,f]azepine *N*	0.015	1.008	0.611	0.100	0.316	0.294	0.698
10,11-dihydro-5*H*-dibenzo[a,d][7]annulene *C10*	0.003	1.247	0.642	0.111	0.333	0.296	0.727
10,11-dihydro-5*H*-dibenzo[a,d][7]annulene *C11*	0.001	1.235	0.693	0.098	0.314	0.268	0.806
10,11-dihydro-5*H*-dibenzo[a,d][7]annulene *C5*	0.011	0.930	0.627	0.035	0.186	0.137	0.644
**C10C11 bond length**							
5*H*-dibenzo[b,f]azepine *C10=C11*	1.240	1.473	1.35	0.001	0.034	0.023	1.341
5*H*-dibenzo[a,d][7]annulene *C10=C11*	1.252	1.499	1.371	0.002	0.044	0.040	1.348
10,11-dihydro-5*H*-dibenzo[b,f]azepine *C10-C11*	1.332	1.606	1.519	0.001	0.038	0.022	1.524
10,11-dihydro-5*H*-dibenzo[a,d][7]annulene *C10-C11*	1.299	1.711	1.524	0.002	0.041	0.026	1.526

**Table 3:** Geometry parameters for optimized structures.

a substituent in the 5-position	**5*H*-dibenzo[b,f]azepine**					**5*H*-dibenzo[a,d][7]annulene**				
	α	C10C11 bond length	distances of the atoms from the A plane			α	C10C11 bond length	distances of the atoms from the A plane		
			C10	C11	N5			C10	C11	C5
unsubstituted structure	45.37	1.345	0.442	0.442	0.511	56.79	1.351	0.527	0.527	0.688
CH_3_	50.49	1.349	0.449	0.449	0.592	51.23	1.349	0.439	0.439	0.636
CH_2_CH_3_	51.46	1.349	0.452	0.456	0.605	51.41	1.349	0.440	0.436	0.640
C(CH_3_)_3_	52.89	1.349	0.498	0.498	0.606	46.15	1.346	0.380	0.380	0.576
CHO	50.80	1.348	0.480	0.483	0.587	54.38	1.350	0.493	0.500	0.662
COOH	52.73	1.350	0.489	0.504	0.612	55.35	1.350	0.512	0.514	0.666
NO_2_	50.42	1.351	0.476	0.476	0.582	52.08	1.350	0.494	0.494	0.614
NH_2_						52.45	1.350	0.450	0.447	0.653
OH						53.35	1.352	0.466	0.466	0.659
Cl						47.70	1.348	0.440	0.440	0.564
a substituent in the 5-position	**10,11-dihydro-5H-dibenzo[b,f]azepine**					**10,11-dihydro-5*H*-dibenzo[a,d][7]annulene**				
	α	C10C11 bond length	distances of the atoms from the A plane			α	C10C11 bond length	distances of the atoms from the A plane		
			C10	C11	N5			C10	C11	C5
unsubstituted structure	22.02	1.544	0.464	0.470	0.003	55.90	1.538	0.891	0.213	0.614
CH_3_	51.82	1.534	0.885	0.143	0.523	43.34	1.534	0.759	0.046	0.480
CH_2_CH_3_	51.79	1.536	0.856	0.151	0.534	51.59	1.536	0.838	0.125	0.571
C(CH_3_)_3_	56.55	1.536	0.232	0.911	0.582	41.90	1.529	0.698	0.053	0.481
CHO	54.37	1.537	0.211	0.901	0.558	49.30	1.535	0.082	0.828	0.542
COOH	57.06	1.539	0.911	0.271	0.597	53.88	1.536	0.180	0.876	0.585
NO_2_	53.94	1.539	0.897	0.227	0.551	50.59	1.535	0.858	0.155	0.530
NH_2_						44.95	1.535	0.774	0.022	0.498
OH						51.59	1.536	0.838	0.125	0.571
Cl						45.09	1.534	0.812	0.047	0.463

**Table 4:** Geometry parameters for carbamazepine structures from CSD crystal database.

Refcode.	Bond Length C10-C11	Distance C10 to the A Plane	Distance C11 to the A Plane	R-factor	T	Space Group
CBMZPN01 [37]	1.330	0.543	0.514	3.5	rt	P21/c
CBMZPN02 [38]	1.325	0.515	0.556	8.4	rt	P21/n
CBMZPN03 [39]	1.3456	0.549	0.540	6.9	rt	R-3
CBMZPN10 [40]	1.331	0.520	0.546	3.9	rt	P21/n
CBMZPN11 [41]	1.337	0.528	0.536	5.06	158	P-1
CBMZPN12 [42]	1.340	0.492	0.469	3.57	158	C2/c
CBMZPN13 [43]	1.376	0.547	0.531	17.96	160	P-1
CBMZPN14 [44]	1.336	0.543	0.518	4.04	rt	P21/n
CBMZPN16 [45]	1.347	0.505	0.571	4.5	123	Pbca
CBMZPN17 [46]	1.350	0.542	0.509	4	rt	P21/n
CBMZPN18 [46]	1.352	0.543	0.510	1.08	100	P21/n
CBMZPN19 [46]	1.352	0.543	0.510		0	P21/n
CBMZPN20 [47]	1.333	0.539	0.527	3.95	rt	P21
CBMZPN21 [48]	1.353	0.545	0.509	6.79	100	P21/n
CBMZPN22 [48]	1.351	0.543	0.510	4.07	100	P21/n
CBMZPN23 [48]	1.352	0.545	0.512	2.4	100	P21/n
CBMZPN27 [49]	1.344	0.511	0.543	4.26	183	P21/n
CBMZPN28 [50]	1.243	0.650	0.514	25.45	rt	P21/n
CBMZPN29 [50]	1.344	0.430	0.490	40.54	rt	P21/n
CBMZPN30 [50]	1.260	0.451	0.452	36.9	rt	P21/n
CBMZPN31 [51]	1.396	0.483	0.545	19.21	rt	P21/n
CBMZPN32 [52]	1.338	0.347	0.395	43.85	rt	P21/n

## Data Availability

Xyz or wfn files are available on request from the corresponding author.

## Sample Availability

Samples of the molecular geometry are available from the authors.
